# Molecular modeling of zinc paddlewheel molecular complexes and the pores of a flexible metal organic framework

**DOI:** 10.1007/s00894-016-2949-5

**Published:** 2016-03-15

**Authors:** Khalid A. H. Alzahrani, Robert J. Deeth

**Affiliations:** Inorganic Computational Chemistry Group, University of Warwick, Coventry, CV4 7AL UK; School of Chemistry, Joseph Black Building, David Brewster Road, Edinburgh, Scotland EH9 3FJ UK

**Keywords:** Force field, Metal organic framework, Molecular dynamics, Molecular mechanics, Transition metal complex

## Abstract

A new all-atom first-principles force field (FF) is constructed for the bimetallic, four-bladed zinc paddlewheel (ZPW) motif. Zinc-ligand interactions are described via Morse functions and the angular geometry at the metal centers is modeled with a pure ligand-ligand repulsion term. The ZPW-FF is principally based on 15 DFT-optimized model systems of general formula ZnPR.nL, where ZnP is the base Zn_2_(O_2_CR)_4_ unit, R = H, CH_3_ or CF_3_, L = NH_3_ or pyridine, and *n* = 0, 1 or 2. It correctly generates the distorted tetrahedral coordination of the uncapped [Zn_2_(O_2_CR)_4_] species in their ground states as well as giving reasonable structures and energies for the higher symmetry D_4h_ transition state conformations. The zinc-ligand Morse function reference distance, *r*_*0*_, is further refined against 30 complexes located in the Cambridge Structural Database and this FF is applied to pore models of the flexible metal-organic framework (MOF) [Zn(bdc)_2_(dabco)]_n_ (bdc = 1,4-benzendicarboxylate; dabco = 1,4-diazabicyclo(2.2.2)octane). A single pore model reproduces the unit cell of the evacuated MOF system while a 3×3 grid model is necessary to provide good agreement with the observed pronounced structural changes upon adsorption of either dimethylformamide or benzene.

**Electronic supplementary material:**

The online version of this article (doi:10.1007/s00894-016-2949-5) contains supplementary material, which is available to authorized users.

**Graphical Abstract:**
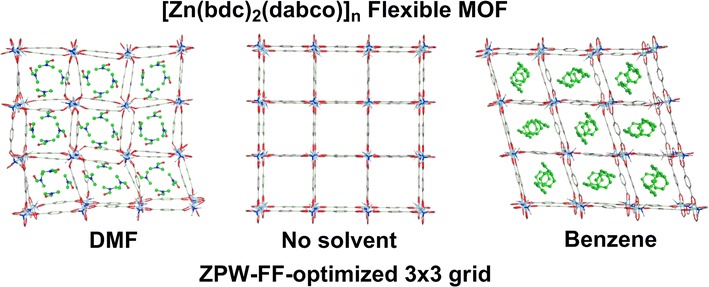
Structural simulations of a zinc paddlewheel metal organic framework reproduce the observed breathing behaviour

## Introduction

Metal organic frameworks (MOFs) are porous materials with a remarkable range of potential applications [1–3]. The framework comprises combinations of secondary building units (SBUs) connected by linkers which can generate a remarkable array of 3-dimensional networks. The SBUs are transition metal complexes or clusters while the linkers are typically organic carboxylates often in combination with polytopic nitrogen-donor ‘pillar’ ligands such as, for example, 1,4-diazabicyclo(2.2.2)octane (dabco) or pyrazine.

While many MOFs have relatively rigid frameworks which therefore define a fixed pore size, other MOFs display a degree of flexibility or ‘breathing’ [4, 5]. The pore size and/or shape changes as a function of adsorbate offering exciting possibilities for using these materials in separations [6–9] and sensing [10, 11].

Some flexible MOFs contain a paddle-wheel SBU. The paddle-wheel motif is a TM dimer bridged by three or four carboxylate units. In combination with linear linkers, the latter generates planar [M_2_L_2_]_n_ grids which can be interconnected by ditopic pillars like dabco to generate a 3-D framework. The classic example is the MOF [Zn_2_(bdc)_2_(dabco)]_n_ (bdc = 1,4-benzenedicarboxylate) which displays a remarkable degree of flexibility depending on the adsorbate _[12]._

**Fig. 1:**
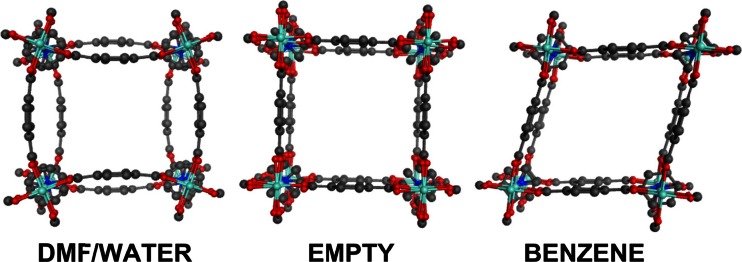
Pore framework structures for [Zn_2_(bdc)_2_(dabco)]_n_ derived from published CIF files [12]. Hydrogens and encapsulated solvent removed. Dabco and carboxylate disorder as per CIF file

As synthesized, the [Zn_2_(bdc)_2_(dabco)]_n_ pore contains one water and four dimethylformamide (dmf) molecules. The framework-adsorbate interactions lead to a pronounced bending deformation of the pore bdc edges (Fig. 1, left) but this disappears on evacuation leaving a more regular cuboidal pore (Fig. 1, middle) which further distorts (and contracts) to a rhombohedral structure upon adsorption of benzene (Fig. 1, right).

Computer modeling of MOFs can provide important atomic level insights into their structures and properties but the structural changes of the type shown in Fig. 1 are computationally challenging and demand a sophisticated theoretical method [13]. Quantum chemical (QC) approaches such as density functional theory (DFT) are fairly general, reasonably accurate and give satisfactory results for a wide range of transition-metal systems. However, MOFs are relatively large and QC is computationally expensive, especially if dynamical properties are of interest [13]. Nevertheless, ab initio molecular dynamics (AIMD) is possible and has been applied, for example, to the breathing of MIL-53(Sc) [14]. Despite the short simulation times of only a few ps, impressive agreement with experiment was obtained. Given its general applicability, we can anticipate many more QC and AIMD studies in the future. Meanwhile, modeling very large systems for a long time, or carrying out virtual high throughput screening,[15] remains the province of classical simulation techniques.

Classical methods such as grand canonical Monte Carlo (GCMC) have been widely employed to model the thermodynamics of adsorbate-MOF interactions [16, 17] but these generally assume a fixed framework which may be inappropriate in the case of flexible MOFs. The alternative is to consider fully atomistic molecular mechanics/molecular dynamics (MM/MD) simulations.

The critical feature of MM/MD is the underlying force field (FF). Generic FFs like UFF [18] and Dreiding [19] can be applied to MOFs but their performance may be of limited accuracy [20]. Thus, while a universal force field is attractive, it is also an extremely challenging undertaking and most of the recent FF development targeted at MOF systems has involved so-called ‘first principles’ parameterization wherein the FF parameters are derived from quantum-chemically-generated training data [21–23]. Bespoke FFs designed for MOFs such as BTW-FF[24], MOF-FF [22], and UFF4MOF [25] should give better accuracy but perhaps at the expense of development time and transferability—i.e., the FF may only work well for the subset of MOFs on which they are trained. In the case of the flexible MOF NH_2_-MIL-53(Al), Garcia-Perez et al. even argue [26] that a fully flexible FF is unnecessary and that a combination of rigid FF combined with some judicious experimental work is sufficient to predict adsoprtion and diffusion of CO_2_ and methane through this material.

In our work deriving accurate FFs targeted at specific transition-metal/ligand combinations, we have used both experimental and/or quantum chemical data [27–35]. Here, we focus on a particular class of flexible MOFs which incorporate the four-bladed zinc paddlewheel (ZPW) motif and construct a new, specialized valence FF, ZPW-FF, based on molecular ZPW complexes which then automatically captures the types of structural change displayed by [Zn_2_(bdc)_2_(dabco)]_n_ as a function of adsorbate. To achieve this, we consider the DFT-calculated chemistry of simple model ZPW systems including those for which there are no experimental data such as the uncapped ‘bare’ Zn_2_(carboxylate)_4_ unit. The latter has either not been explicitly included in the FF development (BTW-FF [24] and MOF-FF [22]) or the structure employed was not the ground state (UFF4MOF [25]). Our new FF is thus based on a consistent set of theoretical data but is then further refined using the structural chemistry of experimentally characterized, single-ZPW systems to reduce the systematic errors from our chosen DFT protocol. The focus on the local coordination environment of the zinc centers gives the ZPW-FF an unprecedented ability to reproduce subtle variations in bond lengths and bond angles and provides DFT-like accuracy at a small fraction of the computational cost. The good accuracy extends to modeling MOFs and we demonstrate that the structural changes observed for [Zn_2_(bdc)_2_(dabco)]_n_ can be successfully reproduced using non-periodic models of its pores. Significantly, the ZPW-FF is based on single ZPW systems so that the subsequent flexibility of the multiple-ZPW pore models emerges as a natural property *predicted* by ZPW-FF.

## Theoretical methods

All the DFT calculations reported here used the ORCA suite version 3.0.1 [36]. The general protocol employed the Becke-Perdew BP86 functional [37, 38] with Ahlrichs’ def2-SVP basis sets [39]. Condensed phase effects [40] were accounted for using the conductor like screening model (COSMO) [41–43] as implemented in ORCA with water as the solvent. Molecular mechanics optimizations used DommiMOE [44], our extension of the 2011 version of the molecular operating environment (MOE) [45]. The as-distributed Merck molecular force field, MMFF94, (mmff94x.ff) was augmented with additional Zn-L-A angle-bending terms and Zn-L-A-B torsional terms. Ligand field molecular mechanics (LFMM) parameters were defined for the zinc coordination [46]. Zn-L interactions were described via Morse functions and the explicit angle bending terms were replaced by a pure ligand-ligand repulsion term of the form A_LL_/d^n^. For these d^10^ Zn^2+^, there is no ligand field stabilization energy and hence all angular overlap model parameters and spin-pairing terms were set to zero. The MOE and LFMM parameter files and partial-charge-setting scripts are included in the supporting information and are also available from the authors (RJD) upon request [47]. Unless otherwise noted, electrostatic interactions employ a distance-dependent dielectric term with a damped cut-off starting at 8 Å going to zero at 10 Å.

A typical NVT ensemble molecular dynamics annealing protocol was as follows: starting T = 50 K; heat to 330 K in 10 ps, hold for 10 ps, cool to 0 K in 10 ps. The Nosé-Poincaré-Andersen algorithm was employed with a 2 fs time step. Bond lengths to H atoms were frozen. A 0.1 fs temperature damping constant was used with configurations sampled every 0.5 ps.

## Results and discussion

A good force field for coordination compounds relies on a diverse set of training data [48, 49]. Our previous experience with Cu(II) FFs shows that the inherent ‘plasticity’ of the Jahn-Teller active d^9^ center yields sufficient diversity that an accurate FF can be constructed using structural data derived solely from experimental X-ray diffraction studies [50]. This is not quite the case for ZPW systems which, at first sight, all seem remarkably similar.

The Cambridge Structural Database (CSD) is a rich source of experimental structural data. Our initial searches were restricted to a central ZPW motif such that none of the Zn-O(carboxylate) bonds were coded as ‘polymeric’. This search thus excludes the majority of (but not all) ZPW MOFs in favor of compounds with isolated, molecular ZPW units and yielded 77 ZPW structures. The Zn-O(carboxylate) distances do not vary very much and average at 2.04 Å with a standard deviation of ∼0.03 Å.

**Fig. 2:**
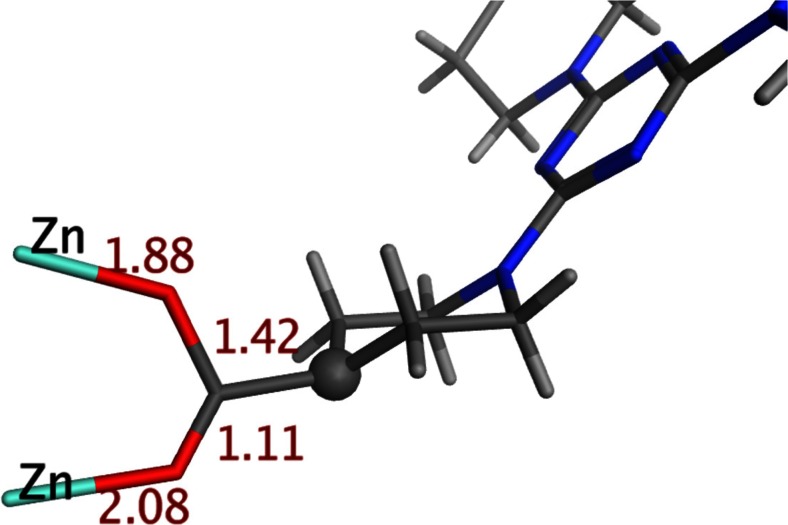
Local detail of carboxylate coordination in WUHHEN. Bond lengths (Å) shown in dark red. The upper Zn-O contact is anomalously short, the carboxylate C-O bonds too asymmetric and one hydrogen is missing off the highlighted carbon (*gray sphere*)

Starting with the extremes, the shortest Zn-O(carboxylate) bond length is reported to be 1.88 Å for catena-(tetrakis (μ_6_−1,1′,1″-(1,3,5-triazine-2,4,6-triyl)tripiperidine-4-carboxylic acid)-hexaaqua-hexa-zinc(II) pyridine dimethyl sulfoxide solvate (CSD refcode WUHHEN) [51]. Although this compound is actually a MOF, the unit cell is sufficiently large to accommodate complete ZPW units and hence pass our test of not having polymeric Zn-O contacts. However, there are a number of anomalous structural features as illustrated in Fig. 2.

The carboxylates are oddly coordinated, the internal C-O distances are very asymmetric and the hydrogen attached to the adjoining carbon atom is not reported in the CIF file. Given that there is significant disorder of incorporated solvent molecules and the overall R factor for the refinement is relatively high (8.4 %), the short Zn-O contact seemed anomalous to us. In any event, this system has water apical ligands and this paper focuses on apical N donor. The extension of ZPW-FF to apical oxygen donors and the anomalous X-ray structure of WUHHEN will be the subject of a future publication.

**Fig. 3:**
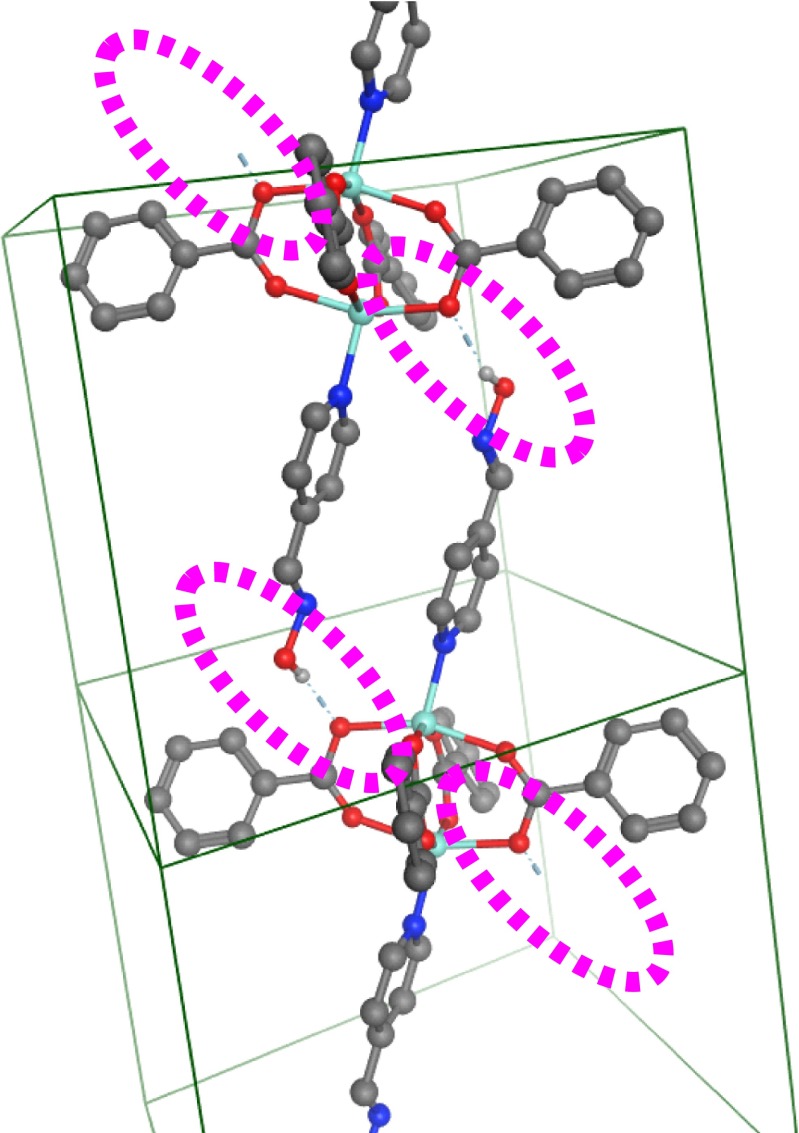
Packing detail for TUFLOW showing intermolecular H-bond contacts (dotted magenta oval) responsible for the long Zn-O distance. (The extra connecting molecules top right and bottom left are omitted for clarity as are all the H atoms bar those involved in the H-bond)

The longest Zn-O bond length in the set of 2.13 Å in (tetrakis(*μ*_2_-benzoato)-bis(pyridine-4-carbaldehyde oxime)-di-zinc(II) (TUFLOW), has a ready experimental interpretation in terms of the intermolecular H-bond between the carboxylate oxygen and the 4-pyridyl-oxime ligand of a neighboring ZPW complex as highlighted in magenta in Fig. 3. This example hints at the sensitivity of the ZPW structure which appears to be relatively easy to distort. This should also provide an exacting test of a force field’s ability to model inter-molecular interactions accurately. However, the initial FF development focuses more on intra-molecular interactions.

Although the overall metal coordination in ZPW system is invariant—every example in the CSD shows five-coordinate Zn centers—there are some subtle variations which a FF should deal with. Given the geometric constraints of the paddle-wheel motif, the local zinc coordination is approximately square pyramidal, especially with nitrogen in the apical position. However, in the absence of electronic effects, as would be expected here for d^10^ Zn^II^ species, five-coordinate complexes should prefer to be trigonal bipyramidal. In addition, pentacoordinate species are well known to be quite flexible and readily undergo Berry pseudorotations. The zinc sites in ZPWs are thus inherently unstable from a mechanical perspective and this manifests itself as a subtle sensitivity of the ZPW to intra- and inter-molecular interactions.

**Fig. 4:**
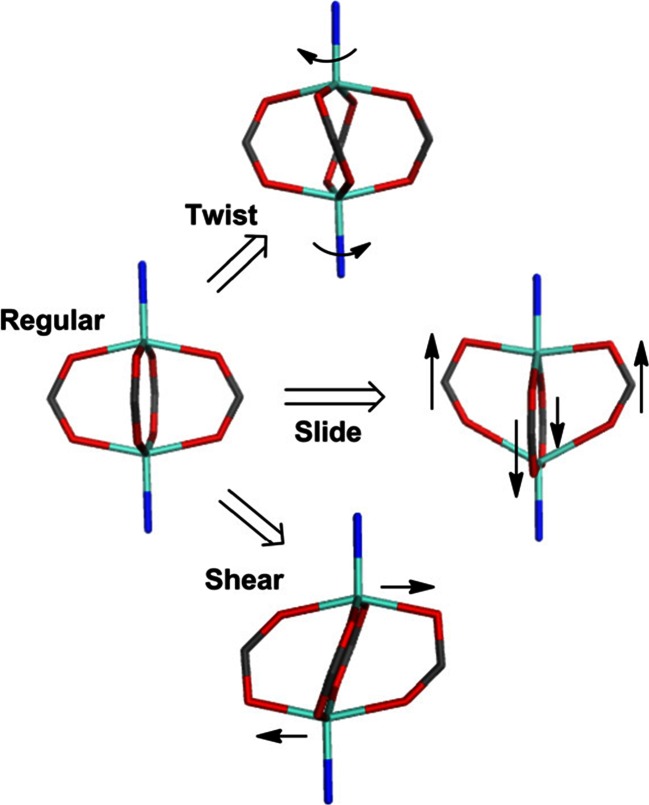
Schematic idealizations of possible distortions of a regular zinc paddlewheel structure

A number of distortions from a regular ZPW structure can be conceived (Fig. 4): (i) a twist around the Zn-Zn vector, (ii) a shear of one ZnO_4_L unit relative to the other, and (iii) a sliding motion of a pair of trans-related carboxylates parallel to the Zn-Zn vector toward one of the zinc centers while the other two carboxylates move in the opposite direction. This makes both Zn geometries more trigonal bipyramidal.

**Fig. 5:**
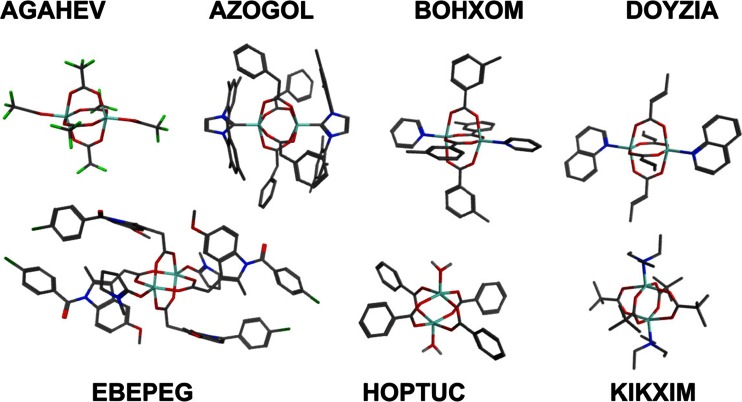
A selection of entries from the CSD used to validate the DFT protocol

Examples of twisted and sheared structures can readily be found in the CSD data but there are apparently no good experimental examples of a pronounced sliding distortion at least with capping N donor ligands. However, such structures can be generated computationally (*vide infra*). DFT is the obvious method and we optimized the structures of a number of ZPW systems both to test the viability of the DFT protocol (BP86/SVP/COSMO(water)) as well as to investigate the source of any apparent distortions in the crystallographic structures. Our choice of functional is based on previous experience [52] plus we note that it was also used for UFF4MOF [25] although the latter employed the ADF program [53], triple-*ζ* STO basis sets and ZORA [54] relativisitic corrections. In any event, a recent benchmarking study suggests that comparable results can be obtained for a wide range of functionals [55] so the particular choice made here is not expected to be especially significant.

As expected, the BP86/DFT/COSMO protocol generally reproduces the experimental structures very well (see Supporting information for overlaid structures). However, the averaged data in Table 1 also show that the chosen DFT methodology systematically overestimates the zinc-ligand distances by around 0.03 to 0.05 Å. The FF will be corrected for this error subsequently.

**Table 1:** Comparison of experimental and calculated bond lengths (Å) for the complexes shown in Fig. 5. The Zn-O(CBX) entry is the averaged Zn-O(carboxylate) distance. Zn-L refers to the bond to the capping group

CSD Refcode	AGAHEV		AZOGOL		BOHXOM		DOYZIA		EBEPEG		HOPTUC		KIKXIM	
	X-ray	DFT	X-ray	DFT	X-ray	DFT	X-ray	DFT	X-ray	DFT	X-ray	DFT	X-ray	DFT
Zn-Zn	3.23	3.23	3.19	3.22	2.98	2.93	2.98	2.96	2.93	2.96	2.98	2.83	2.97	3.05
Zn-O(CBX)	2.05	2.09	2.07	2.10	2.04	2.07	2.04	2.07	2.03	2.07	2.04	2.06	2.03	2.08
Zn–L	1.92	1.97	2.07	2.07	2.04	2.07	2.06	2.09	1.99	2.02	1.99	2.06	2.09	2.15
∆(Zn-Zn)		0.00		−0.03		0.05		0.02		−0.03		0.16		−0.07
∆(Zn-O(CBX))		−0.04		−0.03		−0.03		−0.03		−0.04		−0.02		−0.05
∆(Zn-L)		−0.05		0.00		−0.03		−0.02		−0.03		−0.07		−0.05

The initial FF parameterization is based exclusively on first-principles DFT data. A previous DFT study of zinc and copper paddlewheel systems revealed some interesting structural features [56]. In particular, the ground state for an uncapped ZPW displays a large sliding distortion of D_2d_ symmetry consistent with the four-coordinate Zn centers trying to adopt a tetrahedral geometry. The higher symmetry D_4h_ structure is a transition state. To our knowledge, this feature has not been modeled with any previous FFs for ZPW systems although MOF-FF is formulated in a way which may be able to reproduce the correct ground state [22]. However, the use of an explicit angle-bending term in MOF-FF, even though based on a Fourier series which generates more than one reference angle [57], is not as flexible as the current ZPW-FF approach which has no explicitly angle-bending term at the metal centers and uses instead a 1–3 interaction potential exclusively [58, 59]. Our experience suggests, especially for coordination numbers greater than four, that this should be a better approach compared to methods like UFF4MOF [25] and BTW-FF [24] which have parameters which enforce a particular coordination geometry for a given coordination number. Moreover, the UFF4MOF implementation only considers the higher symmetry transition state structure for the ‘bare’ system plus an explicit Zn-Zn bond is employed which is not physically reasonable but required to generate better structures.

Experimentally, ZPWs all appear to have pentacoordinate metal centers so it is not surprising that no-one has considered a FF for four-coordinate zinc centers. However, this is quite significant for Cu paddlewheel analogues which often display ‘naked’ metal centers plus it also goes toward the inherent flexibility of the MOF.

**Fig. 6:**
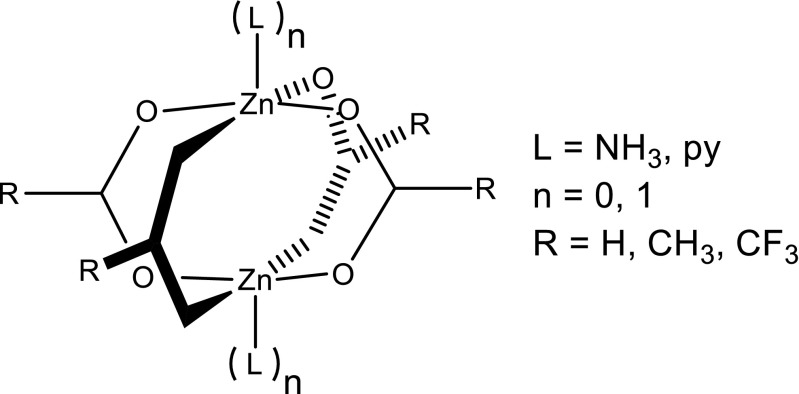
Schematic representation of the ZnPR.nL systems used for initial training

Given that the vast majority of ZPW MOFs have either pyridyl sp^2^ or amine sp^3^ nitrogen capping ligands, the required MMFF94 ligand parameter atom types are NPYD, N, and OX, the latter referring to carboxylate oxygens. Three carboxylates are considered for the basic training species: formate, acetate and trifluoroacetate (R = H, CH_3_ or CF_3_) with zero, one or two capping groups, L, which are either pyridine (py) or ammonia (NH_3_) (Fig. 6). There are thus five complexes for each carboxylate with each species having a general formula ZnPR.nL, where ZnP represents the ⎨Zn_2_(O_2_C)_4_⎬ core of the ZPW. The choice of carboxylates was motivated from a consideration of pK_a_ values. The pK_a_ of acetic acid (4.76) is among the higher values with trifluoroacetic acid (0.23) the lowest. Formate and benzoic acids are intermediate and hence the chosen acids span the relevant pK_a_ range. Benzoic acid is not in the training set but occurs frequently in the validation set (*vide infra*).

Partial atomic charges were modified from the previously published FF for Mn(II) species [60] and are based on superimposing the change in Mulliken charges between uncoordinated and coordinated ligands onto the existing bond charge increment scheme from the Mn(II) FF. The new values are listed in Table 2 along with the standard MMFF94 values for comparison.

**Table 2:** New partial atomic charges for MMFF94 implementation of ZPW-FF. Standard MMFF94 charges in parentheses

Atom type	Environment	New partial charges (standard MMFF94 charge)
OX	All	−0.72 (−0.90)
N	NH_3_	−0.90 (−1.08)
HN	NH_3_	0.43 (0.36)
N	N(Csp^3^)_3_	−0.63 (−0.81)
C	Adjacent to N(Csp^3^)_3_	0.34 (0.27)
NPYD	All	−0.42 (−0.62)
Car	Adjacent to NPYD	0.23 (0.16)
HC	H-C-NPYD	0.23 (0.15)
CO2M	C of formate	0.74 (1.02)
CO2M	C connected to sp^3^ carbon	0.626 (0.906)
Zn + 2	four carboxylates	1.84
Zn + 2	four carboxylates + N	1.45
Zn + 2	four carboxylates + NPYD	1.34

Although there are no d-electron effects for Zn(II), we use the ligand field molecular mechanics (LFMM) method [46, 58] as implemented in DommiMOE,[44] our extended version of the molecular operating environment (MOE) [61]. LFMM parameters for M-L bond stretching (Morse function *r*_0_ and *α*), ligand-ligand repulsion (*A*_LL_), Zn-L-A angle bending (*θ*_0_ and *k*_*θ*_), and Zn-L-A-B torsional twisting (*V*_2_ which favors torsions of 0 and 180°) were manually optimized to minimize the rmsd in Zn-L bonds and heavy-atom (i.e., non-hydrogen) overlays.

**Fig. 7:**
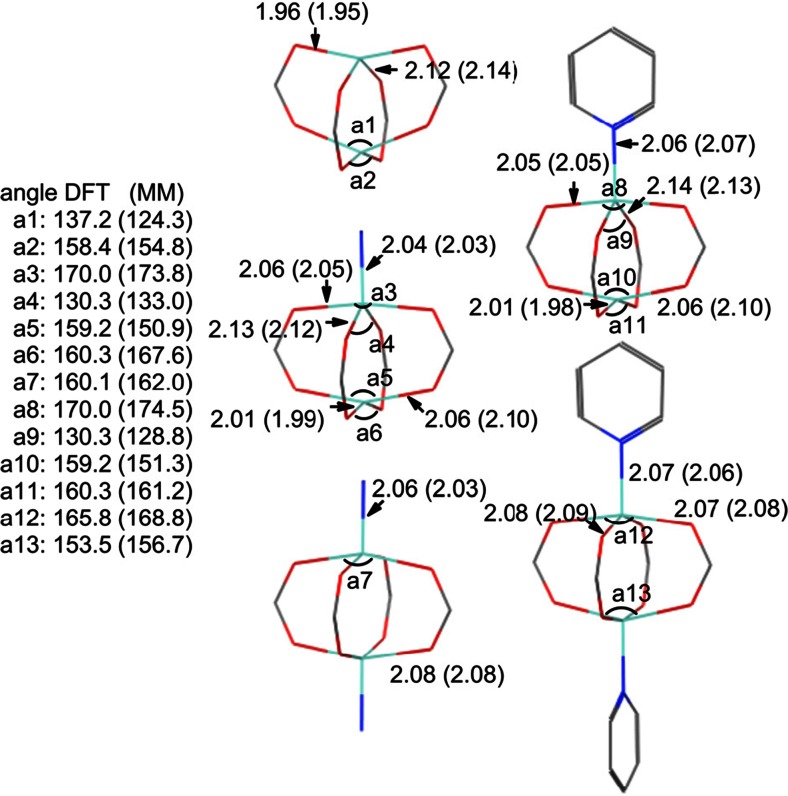
Comparison of optimized DFT and MM (in parentheses) structural parameters for ZnPH.nL, (*n* = 0,1,2; L = NH_3_, py). Only unique Zn-L and L-Zn-L data shown. Distances in Å and angles in degrees. Hydrogens omitted for clarity. Depicted structures are from DFT-optimized coordinates

The resulting ZPW-FF (see Supporting information) reproduces DFT very accurately (see Fig. 7). The overall rmsd in Zn-L distances for all 15 systems is 0.02 Å with the highest value for any one complex being only 0.03 Å. The largest individual error in a single Zn-L bond length is 0.046 Å for two of the Zn-O contacts on the uncapped end of ZnPCF_3_.NH_3_.

The reproduction of the angular geometries is also excellent as evidenced by the overall average rmsd for heavy atom (i.e., non-hydrogen) overlays of only 0.16 Å. The FF successfully captures the broad variation in zinc coordination as well as a number of subtle structural features as illustrated for the formate species in Fig. 7. MM tends to be a little more ‘tetrahedral’ than DFT, but the detailed variation in bond lengths and angles is remarkably well reproduced. This is especially apparent for the lower symmetry species where, for example, the Zn-O distances can vary substantially. In [Zn_2_(O_2_CH)_4_], there are two symmetry independent types of oxygen donor with coordination distances differing by 0.16 Å in the DFT optimization. The ZPW-FF predicts a difference of 0.18 Å. The mono-capped species have an approximately trigonal bipyramidal five-coordinate zinc center in conjunction with a flattened tetrahedral four-coordinate center. Again, the detailed agreement between DFT and ZPW-FF for both bond-length and bond angle variations is excellent.

Using the same ZPW-FF parameters, the structures and relative energies of the transition states for the uncapped species can be compared (Table 3). The structures were optimized using a simple in-house Newton–Raphson code which follows the largest negative eigenvalue. Good starting points for the TS search are needed and these can be conveniently generated by deleting the two capping groups from a ZnPR.2 L system.

**Table 3:** Calculated Zn-O bond lengths (Å), activation energies (kcal mol^−1^) and transition-state frequencies (cm^−1^) for ZnPR transition state systems, R = H, CH_3_, and CF_3_

R	r^‡^ (Zn-O):DFT	r^‡^ (Zn-O):MM	∆E^‡^:DFT^1^	∆G^‡^:DFT^2^	∆E^‡^:MM	*ν* ^‡^:DFT	*ν* ^‡^:MM
H	2.04	2.06	9.4	9.2	6.3	−87	−62
CH_3_	2.03	2.07	10.8	10.3	7.6	−84	−57
CF_3_	2.04	2.06	7.4	7.9	5.3	−60	−44

^a^Raw DFT electronic energy difference

^b^Free energy difference computed using standard statistical mechanics methods implemented in ORCA, *P* = 1 atm, T = 298 K

The ZPW-FF barriers are about 30 % lower than those from DFT but the overall agreement is satisfactory.

**Fig. 8:**
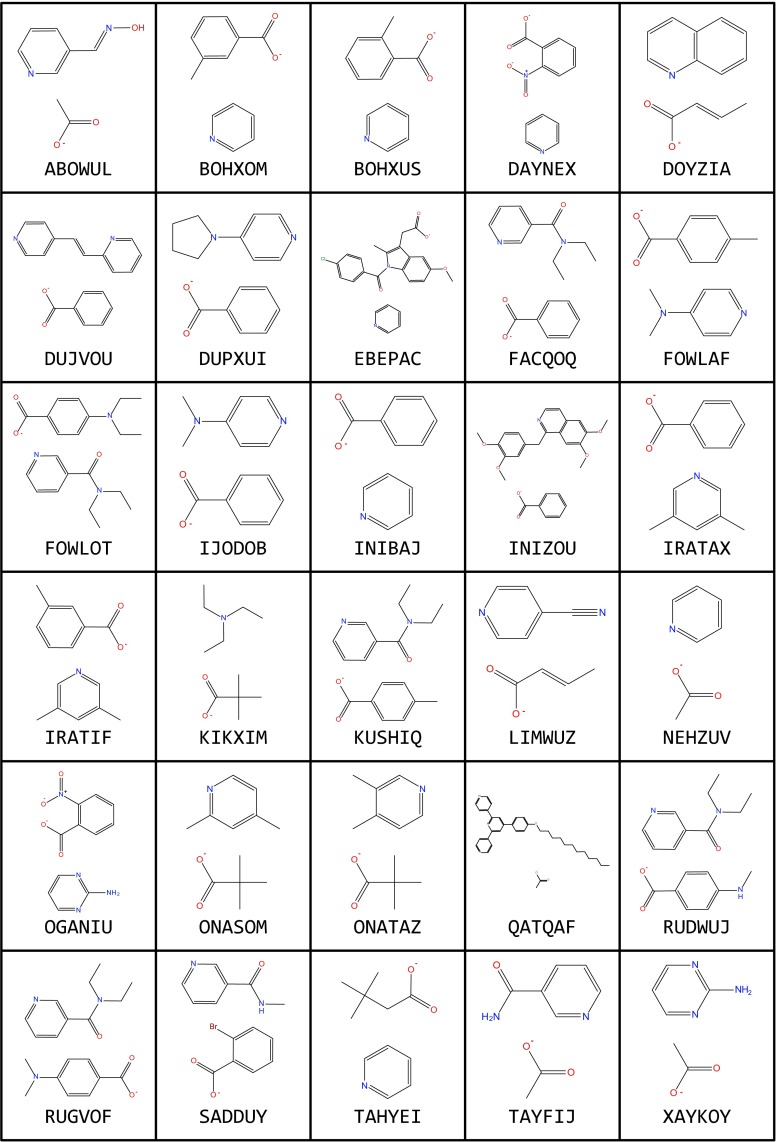
Structural diagrams of ligands for the ZPW systems listed by CSD refcode in Table 4. Only unique combinations displayed. (NEHZUV and NEHHUV01 are the same compound while INIBAJ and QETGAY have the same ligand set)

Recalling the systematic errors between experimental and DFT-optimized complexes, the current FF was retuned using experimental X-ray structural data of 32 ZPW complexes comprising 30 unique ligand combinations (Fig. 8). The refinement was restricted to minor adjustments of Morse *r*_0_ values to minimize the rmsd Zn-L deviations (see Supporting information). The data in Table 4 and in the Supporting information show that the adjusted FF delivers good structural accuracy. The average deviation in zinc-ligand distances is 0.04 Å (Table 4) while 92 % of the individual deviations for all Zn-L distances are less than 0.05 Å and only 1 % of all bond length deviations is larger than 0.1 Å with the largest error being 0.137 Å for XAYKOY (see Supporting information). The overall shapes of the complexes are well reproduced with generally small heavy atom overlay rmsds (Table 4). The latter in particular will be subject to crystal packing influences so we cannot expect (nor want) exact agreement. However, overall, the results give us some confidence to carry on to modeling more complicated systems such as MOFs which contain multiple, interconnected ZPW units.

**Table 4:** Performance of ZPW-FF for molecules shown in Fig. 8. Column 1 CSD refcodes. Column 2, root mean square deviation for Zn-L bond lengths. Column 3, root mean square deviation for heavy atom (i.e., non-hydrogen) overlay. Column 4, difference between experimental and computed Zn-Zn distance (a negative value implies a shorter computed value). All numerical data in Å

Refcode	M-L(rmsd)	Heavy atom rmsd	Zn-Zn diff
ABOWUL	0.03	0.62	−0.07
BOHXOM	0.02	0.30	−0.16
BOHXUS	0.01	0.45	−0.13
DAYNEX	0.01	0.95	−0.21
DOYZIA	0.02	0.45	−0.06
DUJVOU	0.01	0.57	−0.17
DUPXUI	0.04	0.47	−0.13
EBEPAC	0.03	0.87	−0.14
FACQOQ	0.02	1.37	−0.08
FOWLAF	0.04	0.55	−0.17
FOWLOT	0.02	0.75	−0.08
IJODOB	0.04	0.58	−0.15
INIBAJ	0.02	0.29	−0.15
INIZOU	0.02	0.32	−0.15
IRATAX	0.02	0.49	−0.16
IRATIF	0.02	0.59	−0.16
KIKXIM	0.04	0.36	0.05
KUSHIQ	0.03	0.90	−0.16
LIMWUZ	0.03	0.72	−0.12
NEHZUV	0.03	0.29	−0.03
NEHZUV01	0.02	0.13	−0.06
OGANIU	0.04	0.68	−0.12
ONASOM	0.03	0.35	−0.04
ONATAZ	0.03	0.52	−0.04
QATQAF	0.03	0.92	−0.09
QETGAY	0.02	0.26	−0.15
RUDWUJ	0.02	0.75	−0.14
RUGVOF	0.02	0.92	−0.13
SADDUY	0.02	0.93	−0.15
TAHYEI	0.02	0.39	−0.05
TAYFIJ	0.04	0.83	−0.03
XAYKOY	0.05	0.35	−0.04

### Flexible MOFs

Our preliminary investigations into flexible MOF materials focuses on models of individual pores and small grids of pores for [Zn_2_(bdc)_2_(dabco)]_n_. We are motivated by the desire to explore the intimate inter- and intra-molecular interactions both within the framework and between the framework and adsorbed species to assess whether the flexible structures are an inherent feature of the pore. We recognize that many fundamental mechanical, thermal, and dielectric properties will not be available [24] and that care will be required when translating our findings into systems which, in reality, are fully periodic. Nevertheless, we believe the results reported below are significant both in their own right and in terms of a future development of a fully periodic implementation in our DL_POLY_LF platform [62]. Meanwhile, we consider the various ZPW systems highlighted in Fig. 1.

**Fig. 9:**
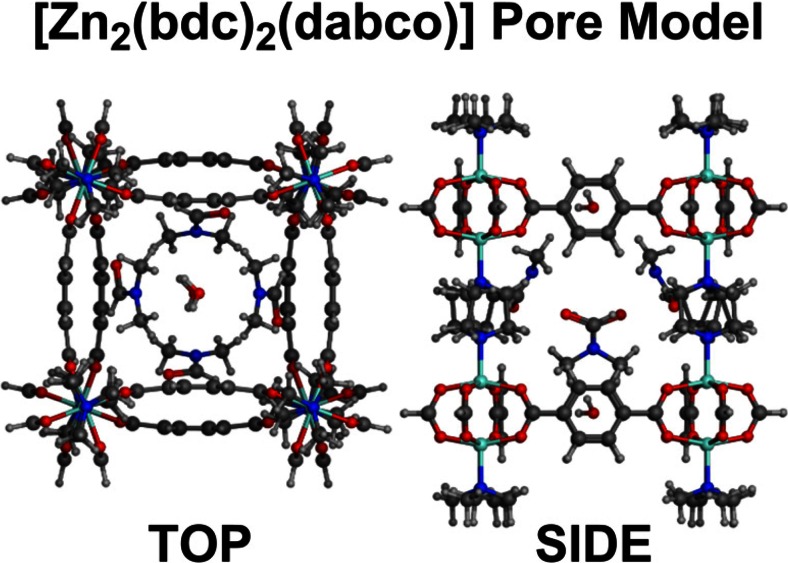
Starting pore model for MM optimizations derived from X-ray diffraction study. Positions of experimental water molecules are shown but waters are not included in the MM calculations

A model for a single pore was constructed from the crystallographic CIF file. It comprises eight ZPW units at the pore corners with dabco pillars. Corner carboxylates are capped with hydrogen while corner zinc centers are capped with Me_3_N groups. The X-ray structure shows significant disorder which results in the dabco groups not having threefold symmetry and the orientations of the dmf carbonyl oxygens is ambiguous. We have selected an arrangement such that the carbonyl oxygen of one dmf is directed toward the HC(O) hydrogen of a neighboring dmf. Water molecules are also identified in the crystal structure but their position at the face of the pore would make them susceptible to effects from adjoining pores. Also, preliminary calculations showed that they have strongly directional H-bonding effects and tend to migrate to the nearest ZPW unit causing significant local distortions. This is one of the shortcomings of using an aperiodic system and the water molecules have therefore not been inclued in the subsequent MM optimizations. Also, given that there are now multiple ZPW units as apposed to the single ZPW systems considered so far, the standard 8 Å/10 Å electrostatic cut-offs are disabled and the default distance-dependent (1/*ε*r^2^) dielectric model for electrostatics is replaced by the softer reaction field implemented in MOE (see Supporting information). Both these changes have minimal effects on either the training systems or the refinement/validation set. The final single-pore model is shown in Fig. 9.

**Fig. 10:**
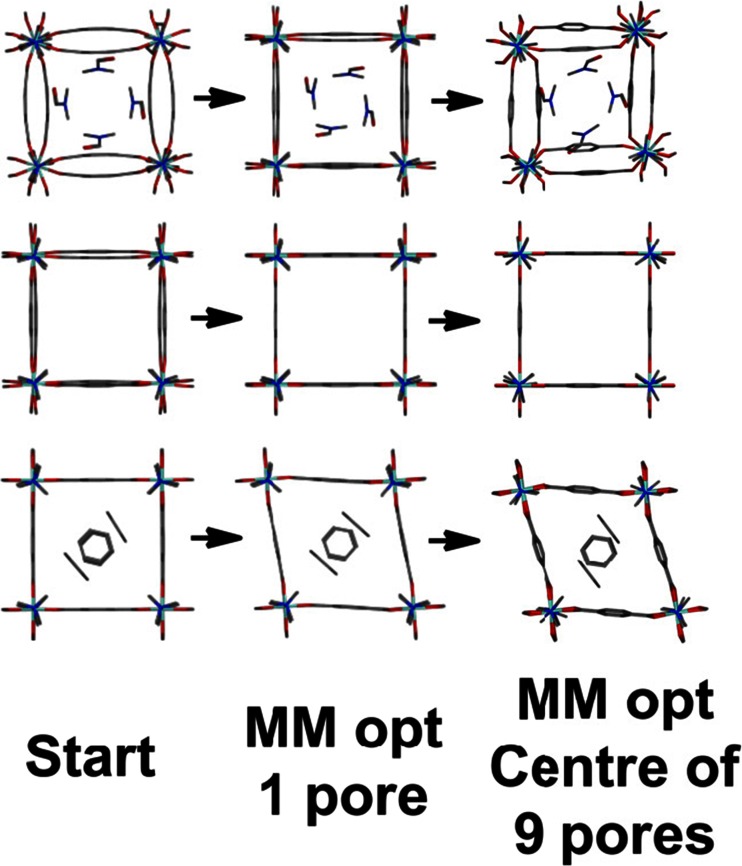
Development of the ‘breathing’ for [Zn(bdc)_2_(dabco)]_n_.solvate. Left column displays the single pore starting model for MM optimization. Central column shows the ZPW-FF optimized structure of the single pore model. The right column shows the ZPW-FF optimized structure of the central pore of the nine-pore 3×3 grid

MM optimizations of the single-pore model with four dmf molecules incorporated display a definite curvature of the pore edges (Fig. 10, middle top). Removal of the dmf molecules leads to a regular ‘cuboidal’ geometry while replacement of the dmf by benzene molecules gives a pronounced rhombohedral distortion. The MM-optimized pores are thus consistent with experiment but, apart from the vacant pore, the calculated distortions are underestimated. In practice, each pore vertex is connected to another pore and it is probably not surprising that a single-pore model is inadequate. Hence, we extended the model to a 3×3 grid of nine pores so that the central one is connected on all sides.

As shown on the right side of Fig. 10, this leads to substantially better agreement with the reported X-ray structural data. The calculated unit cell dimensions of a = 11.0 Å and c = 9.6 Å for the empty pore are virtually identical to the experimental values of 10.9288(15) and 9.6084(12) while for the dmf adsorbate system, the dihedral twist between two ZPW groups connected by a dabco pillar increases from ∼9° for the 1-pore model to ∼30° for the 9-pore model compared to the experimental value of 40°. This twist is accommodated in the modeling largely by a hinge movement along the O-O vector of the carboxylates which keeps the bdc unit flat as opposed to the X-ray structure which shows a smoother curved profile of the bdc bridge. For the benzene-solvated system, the acute angle formed from, for example, the top three zinc centers at the vertices of the pore changes from 81° for the 1-pore model to 75° for the 9-pore model compared to 77° from the X-ray structure.

The current FF thus appears to capture the breathing modes of this particular flexible MOF quite well using an aperiodic model system which we believe is a significant achievement given that only single-ZPW unit systems were employed in its construction. However, part of this success is undoubtedly due to the pillared structure, which means we only need to consider a single layer, plus the positions of the solvent molecules have been taken from experiment. A greater test would be to see whether the number and position of solvent molecules within the pore might be predicted.

We start with benzene since it is more symmetrical than dmf and the experimental structure is not disordered. A single benzene molecule was placed at the center of an empty 1-pore model and energy minimized. It spontaneously migrated to the methylene units of a dabco pillar. Adding a second benzene to this structure, again placed at the center of the pore, and energy minimizing led to the second benzene spontaneously migrating to the opposite dabco pillar. Adding a third benzene to this model, which clearly generates a highly strained starting point, spontaneously leads to the third benzene migrating to the pore surface to form van der Waals contacts with bdc linker aromatic rings. So far, the benzene groups have positioned themselves exactly where the experiment suggests and there is a clear visual indication of where the next benzene should go. However, following the same computational protocol, energy minimization once a fourth benzene is added leads to two results. Sometimes, the fourth benzene goes to the pore face opposite the third and the experimental solid-state packing is realized. Sometimes, the fourth benzene goes to the same face as the third and pushes the latter out such that it interacts with the NMe_3_ groups on the pore periphery. In a periodic system, this benzene would be replicated near the ‘vacant’ face and, once again, we would deduce that this is the more favorable position and the experimental packing would be recovered once again. Visual inspection of a space-filling representation confirms that there is insufficient space to accommodate a fifth benzene. Finally, a number of short 300 K MD annealing simulations were run which confirm that the four benzenes are located in reasonably stable positions.

A similar energy minimization procedure was followed for dmf and, to a large extent, the dmf molecules spontaneously migrated to their experimentally-observed positions. However, while in general, the dmf methyl groups are oriented toward the bdc phenyl rings with the carbonyl groups oriented toward dabco pillars, other orientations of dmf molecules have very similar energies and the barrier to rotation is low.

To test the modeling further, a fifth dmf molecule was added. Energy minimization leads to the fifth dmf being captured in a local minimum within the pore. However, a short MD simulated-annealing run shows that even the 1-pore model cannot sustain five dmf molecules and one is spontaneously pushed out through the relatively unrestricted face formed by bdc linkers. However, in the process, the 1-pore model becomes very badly distorted.

**Fig. 11:**
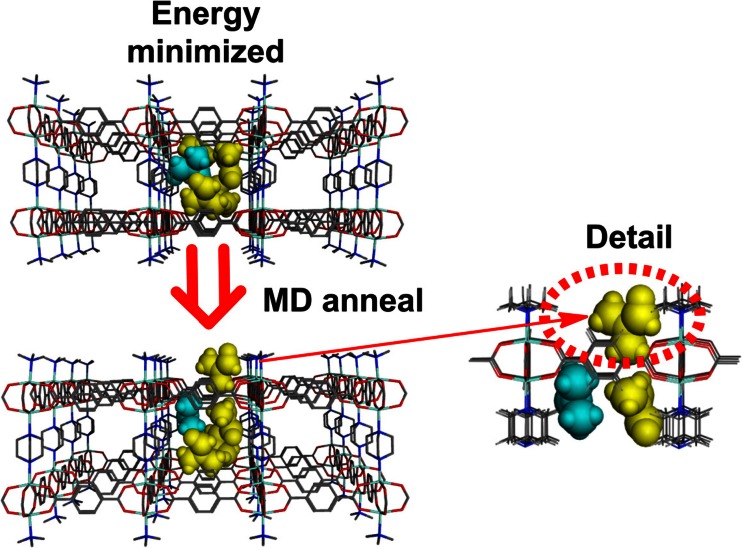
ZPW-FF simulated annealing starting from the energy-minimized, 9-pore model with the central pore containing the four original dmf molecules (*in yellow*) plus a fifth dmf (*in cyan*). After simulated annealing (*bottom left*), one of the dmf molecules exits the pore (*right*, *highlighted by the dotted red ellipse*)

Consequently, we took the 9-pore model and removed the dmf molecules from all but the central pore. A fifth dmf was then added, the energy minimized and a short MD annealing run carried out. As for the 1-pore model, one of the dmfs is spontaneously ejected from the pore (Fig. 11).

**Fig. 12:**
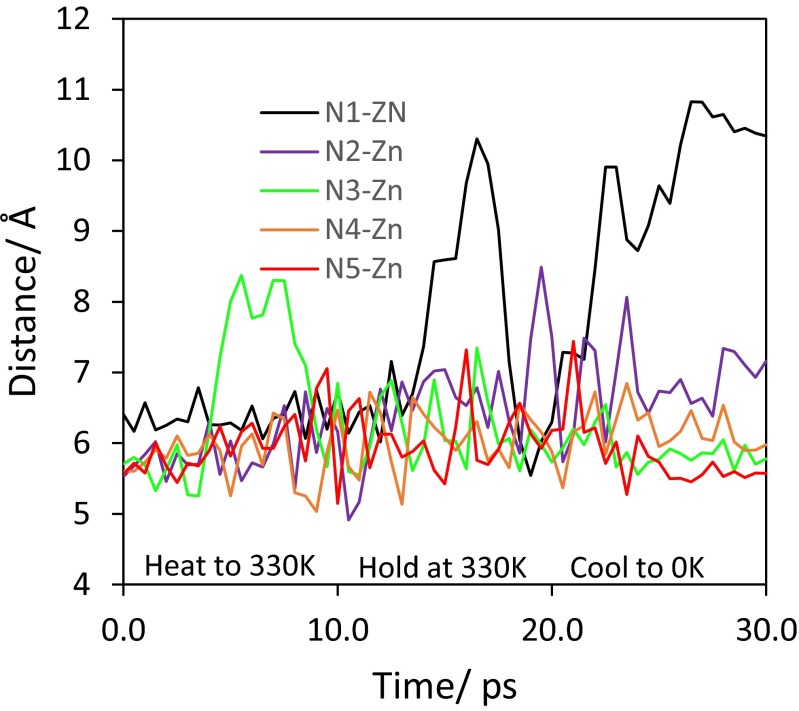
Plot of distances from a corner zinc center to the N atom of the five dmf molecules in the central pore of the 3×3 grid during the simulated annealing MD run showing how one of the dmf molecules (N1) is spontaneously ejected from the central pore

The motions of the dmf molecules are displayed graphically in Fig. 12 which shows the change in the distances between the amide N atoms of each dmf molecule and a nearby Zn atom. Three dmf molecules barely move while N1 is the one which is ejected. N2 adjusts its position to generate the approximately tetrahedral arrangement of dmf molecules in the pore, consistent with the experimental arrangement.

A shortcoming of the preceding series of calculations is the lack of any energetic information. Thus, while we can derive an idea of the maximum loading of adsorbate that an isolated pore can tolerate, we cannot predict the energetic consequences of this loading in the actual MOF since we do not include a full treatment of the pore surroundings. Thus, we cannot deal with the effect of, for example, the external concentration of adsorbate. This requires a more sophisticated dynamics treatment along the lines of that reported by Grosh and Paesani [63]. However, the present study at least demonstrates that the underlying ZPW-FF should provide an excellent platform for such studies and we will report the results in due course.

## Conclusions

A new experimentally-refined, first-principles force field has been developed for the zinc paddlewheel motif including pyridyl and amine capping groups. The ZPW-FF parameters are based on the Merck molecular FF (MMFF) extended with additional ligand field molecular mechanics (LFMM) parameters as implemented in DommiMOE, our extended version of the molecular operating environment. Given the absence of explicit d-electron effects for d^10^ Zn(II) centers, the parameters could easily be ported to other software codes which support the MMFF and LFMM potential energy functional forms.

The new ZPW-FF is based on a much larger set of training and validation systems than other MOF FFs. It accurately reproduces the DFT-computed structures for small model ZPW systems including uncapped, mono-, and di-capped complexes. This includes the first ever empirical modeling of the high-symmetry transition state structures for the uncapped [Zn_2_(O_2_CR)_4_] systems. With a minor refinement of Morse function reference distances for Zn-O and Zn-N bonds, excellent agreement with the structures of 32 crystallographically-characterized ZPW systems is also obtained.

The ZPW-FF is designed to accurately model the local structure of zinc centers coordinated to bridging carboxylate and monodentate N donor ligands and can be applied equally well to isolated ‘molecular’ ZPW systems or to materials such as MOFs with multiple ZPW units. This permits us to construct aperiodic models of MOF pores and explore issues such as whether the behavior observed in the bulk is a function of an individual pore or a set of pores.

The new force field was therefore applied to pore models of the archetypal flexible MOF [Zn_2_(bdc)_2_(dabco)]_n_. The dimensions of the calculated structure of an empty single pore are identical to the experimental unit cell parameters of the extended solid. The framework structure is also sensitive to adsorbed solvent and a 1-pore model already gives a qualitatively correct picture of the effect of including four dmf or four benzene molecules. The latter calculations start from the X-ray crystallographic coordinates but the number and orientation of solvent molecules is also predicted by systematically adding solvent molecules to the pore, energy minimizing and then, if necessary, carrying out short annealing MD runs. The four benzene molecules essentially occupy their experimental positions spontaneously. The dmf molecules show some variability in orientation but the energetic consequences are minor. MD simulations further show that any attempt to add a fifth dmf molecule to the pore will result in one being spontaneously ejected.

Overall, the ZPW-FF performs extremely well, at least for [Zn(bdc)_2_(dabco)]_n_, and is obviously many orders of magnitude more efficient than DFT. Our next goals are to extend the ZPW-FF to other more complicated MOFs and to develop our own version of a copper paddlewheel force field (CPW-FF) where the important d-electron effects arising from the strongly Jahn-Teller active d^9^ copper(II) centers will be captured explicitly by the angular overlap model parameters of LFMM [58, 64] rather than via conventional FF parameters [20]. Also, while pore models may provide some useful insights, the lack of periodicity means that many important mechanical, thermal and dielectric properties cannot be computed [24]. The LFMM methodology has recently been ported to Tinker [65]. The latter avoids the annual licence fees associated with MOE and will hopefully lead to wider uptake by the academic community although there is much development work to be completed before the Tinker-LF implementation can match the functionality currently available in DommiMOE. On the other hand, the ability to model periodic systems in MOE is limited so it is worth noting that LFMM is also available in DL_POLY, although our application involved Pt binding to DNA [62]. We will report on the performance of ZPW-FF for periodic systems in due course.

## Electronic supplementary material

Supporting information

MOE and LFMM parameter files for initial training set. SVL script for setting partial atomic charges. Overlays of X-ray (yellow) and DFT-optimized (blue) structures for selected ZPW systems. Refined ZPW-FF LFMM parameters after recalibration using crystallographic structural data. Detailed structural comparison of ZPW complexes. Form of the electrostatic potential energy employed in MOE.

ESM 1 (DOCX 4249 kb)
