# Supplementary material for: Molecular modeling of zinc paddlewheel molecular complexes and the pores of a flexible metal organic framework
Source: J Mol Model. 2016 Mar 15;22:80. doi: 10.1007/s00894-016-2949-5 (PMC4792333; doi:10.1007/s00894-016-2949-5)
Supplement: Supplementary file 1 — (DOCX 4249 kb) [file 894_2016_2949_MOESM1_ESM.docx]

Submitted to: Journal of Molecular Modeling

Supporting Information

Molecular Modeling of Zinc Paddlewheel Molecular Complexes and the Pores of a Flexible Metal Organic Framework

Khalid A. H. Alzahrani and Robert J. Deeth*^†^
Inorganic Computational Chemistry Group
University of Warwick
Coventry CV4 7AL, UK

^†^ Current address: School of Chemistry, University of Edinburgh

**Contents:**

MOE and LFMM parameter files for initial training set.

SVL script for setting partial atomic charges.

Overlays of X-ray (yellow) and DFT-optimised (blue) structures for selected ZPW systems.

Refined ZPW-FF LFMM parameters after recalibration using crystallographic structural data.

Detailed structural comparison of ZPW complexes.

Form of the electrostatic potential energy employed in MOE.

**MOE and LFMM parameter files for initial training set**

# LFMM parameters for MMFF94 Zinc Paddlewheels

# rjd: Sep 2015 TRAINING SET

[Morse]

#T1 T2 dist D a

Zn+2 OX 1.95 50.7 1.65 0.0 0.0

Zn+2 N 1.99 58.0 1.40 0.0 0.0

Zn+2 NPYD 1.98 60.0 1.40 0.0 0.0

%

[ll]

#M L A n

Zn+2 OX 6700 6

Zn+2 N 5500 6

Zn+2 NPYD 3000 6

%

[esig]

#M L esig0 esig1 esig2 esig3 esig4 esig5 esig6

Zn+2 OX 0 0 0 0 0 0 0

Zn+2 NPYD 0 0 0 0 0 0 0

Zn+2 N 0 0 0 0 0 0 0

%

[epix]

#M L epix0 epix1 epix2 epix3 epix4 epix5 epix6

Zn+2 OX 0 0 0 0 0 0 0

Zn+2 N 0 0 0 0 0 0 0

Zn+2 NPYD 0 0 0 0 0 0 0

%

[epiy]

#M L epiy0 epiy1 epiy2 epiy3 epiy4 epiy5 epiy6

Zn+2 OX 0 0 0 0 0 0 0

Zn+2 N 0 0 0 0 0 0 0

Zn+2 NPYD 0 0 0 0 0 0 0

%

[exds]

#M L exds0 exds1 exds2 exds3 exds4 exds5 exds6

Zn+2 OX 0 0 0 0 0 0 0

Zn+2 N 0 0 0 0 0 0 0

Zn+2 NPYD 0 0 0 0 0 0 0

%

[pair]

#M L P0 P1 P2 P3 P4 P5 P6

Zn+2 OX 0 0 0 0 0 0 0

Zn+2 N 0 0 0 0 0 0 0

Zn+2 NPYD 0 0 0 0 0 0 0

%

# Additional MMFF94x MOE force field parameters for Zinc Paddlewheels

# rjd Sep 2015: TRAINING SET

type Zn+2 Zn 'Zn+2 (d10)'

[rules]

#transition series metal cations, by row

#first row

Zn+2 match '[Zn+2]' #Zn+2

#if matching fails, use atom names

Zn+2 atom-name 'Zn+2' #Zn+2

#rjd: match bonded ligand types to free ligand types

OH2 match '[OX3]([#T])([#1])([#1])' # water ligand

HOH match '[#1]O([#T])([#1])' # hydrogens of water ligand

N match '[NX4][#T]' # amine ligand

HN match '[#1][NX4][#T]' # H of amine ligand

NPYD match 'n([#T])(c)(c)' # pyridyl nitrogen

[ang] # ------------------------- TM ANGLE PARAMETERS --------------------------

ang-function angle

#code T1 T2 T3 angle k2 k3 k4

* Zn+2 OX CO2M 125.0 24.0 0.0 0.0

* Zn+2 N HN 120.0 60.0 0.0 0.0

* Zn+2 NPYD Car 120.0 10.0 0.0 0.0

0 OX CO2M OX 125.000 100. 0.0 0

[stb] # ------------------- stretch-bend parameters ------------------------

#code T1 T2 T3 kbIJK kbKJI

* OX CO2M Car 0.0 0.0

* Car Car CO2M 0.0 0.0

* Zn+2 OX CO2M 0.0 0.0

* Zn+2 N5B C5A 0.0 0.0

* NPYD Zn+2 OX 0.0 0.0

* OX Zn+2 OX 0.0 0.0

* N Zn+2 OX 0.0 0.0

* Zn+2 NPYD Car 0.0 0.0

* Zn+2 N C 0.0 0.0

[ptor] # ------------------- PROPER TORSIONS -------------------------------

#code T1 T2 T3 T4 V1/2 V2/2 V3/2 V4/2 V5/2

* Zn+2 OX CO2M HC 0.000 -2.500 0.000 0.000 0.000

* Zn+2 OX CO2M OX 0.000 -2.500 0.000 0.000 0.000

* Zn+2 OX CO2M Car 0.000 -1.000 0.000 0.000 0.000

* Zn+2 OX CO2M C 0.000 -2.500 0.000 0.000 0.000

* Zn+2 OX CO2M Csp2 0.000 -2.500 0.000 0.000 0.000

* Zn+2 OX CO2M Car 0.000 -1.000 0.000 0.000 0.000

* Zn+2 NPYD Car Car 0.000 -5.000 0.000 0.000 0.000

[oop] # ------------------- out of plane parameters --------------------------

#T1 T2* T3 T4 koop

Zn+2 NPYD Car Car 2.00

[nonbonded] # ---------- nonbonded atomic parameters ------------------------

#type radius well apol Neff mass DA q0 fcadj pbci

Zn+2 1.620 0.106 0.400 6.000 - - 2.0000 0.0000 0.0000

**SVL script for setting partial atomic charges**

function PartialCharge;

function fix_carboxylates[]

// Detect mononuclear carboxylate, make sure it's not in a chelate ring

// join up both oxygens and check carbon charge

write ['Start of carboxylate fix\n'];

local metkeys = Atoms[] | sm_Match [ '[Zn]', Atoms[] ];

//pr metkeys;

// Set metal names to correct value

aSetName [ metkeys, 'Zn+2'];

aSetIon [ metkeys, 2];

// Fix donor N ionisation states while we're at it...

local n_keys = Atoms[] | sm_Match [ '[#7][Zn]', Atoms[] ];

aSetIon [n_keys, 0];

aSetHintLP [ n_keys, 1];

// Find coordinated bridging carboxylates

local ligkeys = split [ cat sm_MatchAtoms [ 'C(O[#T])(O[#T])', Atoms[] ], 5 ];

local n_carb = length ligkeys;

write ['>>> {n:} bridging carboxylate ligands found\n', length ligkeys ];

// Set HintLP on oxygens, and ionisation states

local il;

for il = 1, length ligkeys loop

aSetHintLP [ ligkeys(il)(2), 1];

aSetHintLP [ ligkeys(il)(4), 1];

aSetIon [ ligkeys(il)(1), 1]; // Carbon

// aSetIon [ ligkeys(il)(2), -1]; // Oxygen

// aSetIon [ ligkeys(il)(4), -1]; // Oxygen

endloop;

// Set force field charges to current FF - CAUTION: only MMFF94_tm seems to work

local [q, pos] = PartialCharge [Atoms[], 'FF'];

aSetCharge [ Atoms[], q ];

local tot_chg = pr (add aCharge Atoms[] - n_carb); // adjust total charge for no. carboxylates

//Adjust carbon and oxygen charges

// Automatic charges make carboxylate charge one unit too positive

local c_alter = -0.78; // rjd 0.22 derived from Khalid's DFT calculations

local o_alter = -0.15; // rjd O charge based on bci

local c_keys = (tr ligkeys)(1);

local o_keys = cat [(tr ligkeys)(2),(tr ligkeys)(4)];

//pr o_keys; exit[];

local c_charge = aCharge c_keys;

local o_charge = aCharge o_keys;

aSetCharge [ c_keys, c_charge + c_alter ];

aSetCharge [ o_keys, o_charge + o_alter ];

// Find bidentate carboxylates

ligkeys = split [ cat sm_MatchAtoms [ 'C1O[#T]O1', Atoms[] ], 4 ];

for il = 1, length ligkeys loop

aSetHintLP [ ligkeys(il)(2), 1];

aSetHintLP [ ligkeys(il)(4), 1];

aSetIon [ ligkeys(il)(1), 1]; // Carbon

aSetIon [ ligkeys(il)(2), -1]; // Oxygen

aSetIon [ ligkeys(il)(4), -1]; // Oxygen

endloop;

c_keys = (tr ligkeys)(1);

o_keys = cat [(tr ligkeys)(2),(tr ligkeys)(4)];

c_charge = aCharge c_keys;

o_charge = aCharge o_keys;

aSetCharge [ c_keys, c_charge + c_alter ];

aSetCharge [ o_keys, o_charge + o_alter ];

// Find water bridges and set H charges to 0.54

ligkeys = split [ cat sm_MatchAtoms [ '[OH2]([#1])([#1])([#T])([#T])', Atoms[] ], 5 ];

o_keys = cat [(tr ligkeys)(1)];

local h_keys = cat [(tr ligkeys)(2),(tr ligkeys)(3)];

o_charge = aCharge o_keys;

local h_charge = first aCharge h_keys;

o_alter = 2* (0.54 - h_charge);

aSetCharge [ h_keys, 0.54 ];

aSetCharge [ o_keys, o_charge - o_alter ];

// Find Zn-NR3 units and make the R atoms 0.07 more positive

//ligkeys = split [ cat sm_MatchAtoms [ 'N([#1])([#1])([#1])([#T])', Atoms[] ], 5 ];

ligkeys = split [ cat sm_MatchAtoms [ 'N(*)(*)(*)([#T])', Atoms[] ], 5 ];

h_keys = cat [(tr ligkeys)(2),(tr ligkeys)(3),(tr ligkeys)(4)];

//h_charge = first aCharge h_keys;

h_charge = aCharge h_keys;

aSetCharge [ h_keys, h_charge + 0.07 ];

// Find Zn-py and adjust C/H charges next to N

ligkeys = split [ cat sm_MatchAtoms [ 'n(c[#1])(c[#1])([#T])', Atoms[] ], 6 ];

h_keys = cat [(tr ligkeys)(3),(tr ligkeys)(5)];

h_charge = first aCharge h_keys;

aSetCharge [ h_keys, h_charge + 0.08 ];

//aSetSelected [ h_keys, 1 ]; exit[];

c_keys = cat [(tr ligkeys)(2),(tr ligkeys)(4)];

c_charge = first aCharge c_keys;

aSetCharge [ c_keys, c_charge + 0.07 ];

// Adjust Zinc charges

local chg_diff = tot_chg - (add aCharge Atoms[]);

// Four coord: rho(Zn) = 1.84

// Five coord: ammonia: rho(Zn) = 1.45

// Five coord: pyridine: rho(Zn) = 1.34

local mkey, bkeys;

for mkey in metkeys loop

bkeys = cat aBonds mkey;

if (length bkeys) == 4 then

aSetCharge [ mkey, 1.84 ];

elseif add (aMMType bkeys == 'NPYD' ) == 1 then // !!! MMFF94 specific !!!

aSetCharge [ mkey, 1.34 ];

elseif add (aMMType bkeys == 'N' ) == 1 then

aSetCharge [ mkey, 1.45 ];

endif;

endloop;

//pr chg_diff; exit[];

//aSetCharge [ metkeys, (aCharge metkeys) + (chg_diff/(length metkeys)) ];

write ['>>> Total molecular charge: {n:5.1f}\n', add aCharge Atoms[] ];

endfunction

**Overlays of X-ray (yellow) and DFT-optimised (blue) structures for selected ZPW systems. Hydrogens omitted for clarity.**

**Refined ZPW-FF LFMM parameters after recalibration using crystallographic structural data. Only the Morse function *α* values have been changed relative to the training set parameters.**

# LFMM parameters for MMFF94

#

# LFMM_MMFF94.par

#

# rjd 6/11/2013: swapped Mn+2 for Zn+2

# rjd 22/09/2015: for ZPW pore models

#title 'LFMM'

[Morse]

#T1 T2 dist D alpha

# X-ray fit

Zn+2 OX 1.90 50.7 1.65 0.0 0.0

Zn+2 N 1.99 58.0 1.40 0.0 0.0

Zn+2 NPYD 1.92 60.0 1.40 0.0 0.0

%

[ll]

#M L A n

Zn+2 OX 6300 6

Zn+2 N 5300 6

Zn+2 NPYD 5000 6

%

[esig]

#M L esig0 esig1 esig2 esig3 esig4 esig5 esig6

Zn+2 N 0 0 0 0 0 0 0

Zn+2 NPYD 0 0 0 0 0 0 0

Zn+2 OX 0 0 0 0 0 0 0

%

[epix]

#M L epix0 epix1 epix2 epix3 epix4 epix5 epix6

Zn+2 N 0 0 0 0 0 0 0

Zn+2 NPYD 0 0 0 0 0 0 0

Zn+2 OX 0 0 0 0 0 0 0

%

[epiy]

#M L epiy0 epiy1 epiy2 epiy3 epiy4 epiy5 epiy6

Zn+2 N 0 0 0 0 0 0 0

Zn+2 NPYD 0 0 0 0 0 0 0

Zn+2 OX 0 0 0 0 0 0 0

%

[exds]

#M L exds0 exds1 exds2 exds3 exds4 exds5 exds6

Zn+2 N 0 0 0 0 0 0 0

Zn+2 NPYD 0 0 0 0 0 0 0

Zn+2 OX 0 0 0 0 0 0 0

%

[pair]

#M L P0 P1 P2 P3 P4 P5 P6

Zn+2 N 0 0 0 0 0 0 0

Zn+2 NPYD 0 0 0 0 0 0 0

Zn+2 OX 0 0 0 0 0 0 0

%

**Detailed structural comparison of ZPW complexes.**

| Refcode | Lig name/type | M-  L(LFMM-Xray) |  |  |  |  |  |  |  |  |  |  |  |  |
| --- | --- | --- | --- | --- | --- | --- | --- | --- | --- | --- | --- | --- | --- | --- |
| ABOWUL | NPYD/N1 | OX/O1 | OX/O3 | OX/O2A | OX/O4A | OX/O2 | OX/O4 | NPYD/N1A | OX/O1A | OX/O3A | M-L rms) | Heavy Atom rmsd | Zn-Zn DFT | Zn-Zn LFMM |
|  | -0.013 | -0.017 | 0.067 | 0.002 | 0.041 | 0.002 | 0.041 | -0.014 | -0.016 | 0.067 | 0.036 | 0.393 | 2.923 | 2.895 |
| BOHXOM | OX/O1 | OX/O2 | NPYD/N1 | OX/O1B | OX/O2B | OX/O1C | OX/O1A | OX/O2C | OX/O2A | NPYD/N1A |  |  |  |  |
|  | 0.033 | 0.001 | 0.012 | 0.033 | 0.001 | 0.033 | 0.033 | 0.001 | 0.002 | 0.012 | 0.022 | 0.289 | 2.980 | 2.874 |
| BOHXUS | OX/O1 | OX/O2 | OX/O3 | OX/O4 | NPYD/N1 | OX/O1B | OX/O2B | OX/O3B | OX/O4B | NPYD/N1B |  |  |  |  |
|  | 0.032 | 0.001 | 0.018 | 0.015 | 0.008 | 0.032 | 0.001 | 0.018 | 0.015 | 0.008 | 0.018 | 0.748 | 2.934 | 2.856 |
| DAYNEX | NPYD/N3 | OX/O1 | OX/O2 | OX/O5 | OX/O6 | NPYD/N3A | OX/O1A | OX/O2A | OX/O5A | OX/O6A |  |  |  |  |
|  | 0.001 | 0.034 | 0.013 | -0.007 | 0.000 | 0.000 | 0.034 | 0.013 | -0.006 | 0.000 | 0.017 | 0.952 | 3.030 | 2.870 |
| DOYZIA | OX/O1 | OX/O2 | OX/O3 | OX/O4 | NPYD/N1 | OX/O2B | OX/O4B | OX/O1B | OX/O3B | NPYD/N1B |  |  |  |  |
|  | 0.026 | 0.036 | 0.028 | 0.011 | 0.011 | 0.036 | 0.011 | 0.027 | 0.028 | 0.011 | 0.025 | 0.466 | 2.975 | 2.953 |
| DUJVOU | OX/O1 | OX/O3 | NPYD/N1 | OX/O2D | OX/O4D | OX/O2 | OX/O4 | OX/O1D | OX/O3D | NPYD/N1D |  |  |  |  |
|  | 0.019 | 0.019 | -0.002 | 0.012 | 0.007 | 0.012 | 0.007 | 0.018 | 0.020 | -0.002 | 0.014 | 0.415 | 2.970 | 2.846 |
| DUPXUI | OX/O1 | OX/O3 | NPYD/N1 | OX/O2B | OX/O4B | OX/O2 | OX/O4 | OX/O1B | OX/O3B | NPYD/N1B |  |  |  |  |
|  | 0.003 | 0.022 | 0.078 | 0.022 | 0.006 | 0.022 | 0.007 | 0.003 | 0.022 | 0.079 | 0.038 | 0.473 | 2.982 | 2.902 |
| EBEPAC | OX/O1 | OX/O2 | OX/O5 | OX/O6 | NPYD/N3 | OX/O2A | OX/O1A | OX/O6A | OX/O5A | NPYD/N3A |  |  |  |  |
|  | 0.041 | -0.019 | 0.056 | 0.022 | 0.010 | -0.019 | 0.041 | 0.022 | 0.057 | 0.011 | 0.034 | 0.873 | 2.969 | 2.879 |
| FACQOQ | NPYD/N1 | OX/O2 | OX/O3 | OX/O4 | OX/O5 | OX/O3D | NPYD/N1D | OX/O2D | OX/O5D | OX/O4D |  |  |  |  |
|  | 0.005 | -0.011 | 0.033 | 0.040 | 0.014 | 0.033 | 0.005 | -0.010 | 0.014 | 0.040 | 0.025 | 1.403 | 2.866 | 2.835 |
| FOWLAF | OX/O2 | OX/O3 | NPYD/N1 | OX/O1B | OX/O4B | OX/O1 | OX/O4 | OX/O2B | OX/O3B | NPYD/N1B |  |  |  |  |
|  | -0.004 | 0.024 | 0.073 | 0.002 | 0.035 | 0.002 | 0.035 | -0.004 | 0.023 | 0.073 | 0.038 | 0.328 | 3.029 | 2.901 |
| FOWLOT | OX/O1 | OX/O2 | NPYD/N1 | OX/O3B | OX/O4B | OX/O3 | OX/O4 | OX/O1B | OX/O2B | NPYD/N1B |  |  |  |  |
|  | 0.057 | 0.024 | -0.007 | 0.036 | 0.015 | 0.036 | 0.016 | 0.057 | 0.023 | -0.007 | 0.033 | 0.884 | 2.887 | 2.903 |
| IJODOB | NPYD/N1 | OX/O1 | OX/O2 | OX/O3 | OX/O4 | NPYD/N1B | OX/O1B | OX/O2B | OX/O3B | OX/O4B |  |  |  |  |
|  | 0.079 | 0.022 | 0.022 | 0.021 | -0.020 | 0.078 | 0.022 | 0.022 | 0.021 | -0.020 | 0.040 | 0.582 | 3.004 | 2.899 |
| INIBAJ | NPYD/N1 | OX/O1 | OX/O3 | OX/O4 | OX/O2B | OX/O2 | NPYD/N1B | OX/O1B | OX/O3B | OX/O4B |  |  |  |  |
|  | -0.001 | 0.025 | 0.024 | 0.041 | -0.002 | -0.002 | 0.000 | 0.026 | 0.024 | 0.042 | 0.024 | 0.287 | 2.961 | 2.867 |
| INIZOU | NPYD/N1 | OX/O1 | OX/O2 | OX/O3 | OX/O4 | OX/O2B | NPYD/N1B | OX/O1B | OX/O4B | OX/O3B |  |  |  |  |
|  | 0.038 | 0.036 | 0.004 | 0.004 | 0.021 | 0.004 | 0.038 | 0.036 | 0.021 | 0.004 | 0.026 | 0.379 | 3.053 | 2.948 |
| IRATAX | NPYD/N1 | OX/O1 | OX/O3 | OX/O2A | OX/O4A | OX/O2 | OX/O4 | NPYD/N1A | OX/O1A | OX/O3A |  |  |  |  |
|  | -0.006 | 0.037 | 0.013 | 0.000 | 0.028 | 0.001 | 0.025 | -0.007 | 0.038 | 0.010 | 0.022 | 0.733 | 2.970 | 2.856 |
| IRATIF | NPYD/N1 | OX/O2 | OX/O3 | OX/O1A | OX/O4A | OX/O1 | OX/O4 | NPYD/N1A | OX/O2A | OX/O3A |  |  |  |  |
|  | -0.009 | 0.017 | 0.034 | 0.026 | -0.001 | 0.026 | -0.001 | -0.008 | 0.017 | 0.035 | 0.021 | 0.893 | 2.964 | 2.857 |
| KIKXIM | OX/O1 | OX/O2 | OX/O3 | OX/O4 | N/N1 | OX/O1B | OX/O2B | OX/O3B | OX/O4B | N/N1B |  |  |  |  |
|  | 0.039 | 0.052 | 0.039 | 0.049 | 0.043 | 0.039 | 0.053 | 0.039 | 0.049 | 0.043 | 0.045 | 0.332 | 2.975 | 3.032 |
| KUSHIQ | OX/O1 | OX/O2 | OX/O3 | OX/O4 | NPYD/N1 | OX/O1A | OX/O2A | OX/O3A | OX/O4A | NPYD/N1A |  |  |  |  |
|  | 0.032 | 0.018 | -0.054 | 0.029 | -0.004 | 0.032 | 0.018 | -0.053 | 0.030 | -0.004 | 0.032 | 0.949 | 2.949 | 2.835 |
| LIMWUZ | OX/O1 | OX/O3 | NPYD/N1 | OX/O1G | OX/O3G | OX/O2 | OX/O4 | NPYD/N3 | OX/O2G | OX/O4G |  |  |  |  |
|  | 0.014 | 0.029 | -0.026 | 0.010 | 0.050 | 0.028 | 0.000 | -0.029 | 0.048 | -0.004 | 0.029 | 0.760 | 2.910 | 2.857 |
| NEHZUV | OX/O1 | OX/O3 | NPYD/N1 | OX/O1A | OX/O3A | OX/O2 | OX/O4 | NPYD/N2 | OX/O2A | OX/O4A |  |  |  |  |
|  | 0.034 | 0.020 | 0.000 | 0.035 | 0.020 | 0.048 | 0.019 | -0.008 | 0.050 | 0.019 | 0.030 | 0.286 | 2.892 | 2.898 |
| NEHZUV01 | OX/O1 | OX/O2 | OX/O3 | OX/O4 | NPYD/N1 | OX/O2D | OX/O1D | OX/O4D | OX/O3D | NPYD/N1D |  |  |  |  |
|  | 0.025 | 0.034 | 0.025 | 0.025 | -0.006 | 0.035 | 0.025 | 0.025 | 0.025 | -0.006 | 0.025 | 0.131 | 2.917 | 2.897 |
| OGANIU | OX/O1 | OX/O2 | OX/O7 | OX/O8 | NPYD/N2 | OX/O1B | OX/O2B | OX/O7B | OX/O8B | NPYD/N2B |  |  |  |  |
|  | -0.009 | 0.109 | -0.017 | 0.018 | -0.027 | -0.007 | 0.109 | -0.017 | 0.018 | -0.026 | 0.051 | 0.661 | 3.008 | 2.914 |
| ONASOM | NPYD/N1 | OX/O3 | OX/O4 | OX/O1A | OX/O2A | OX/O1 | OX/O2 | NPYD/N1A | OX/O3A | OX/O4A |  |  |  |  |
|  | 0.011 | 0.032 | 0.034 | 0.041 | 0.034 | 0.040 | 0.033 | 0.012 | 0.034 | 0.033 | 0.032 | 0.411 | 2.958 | 2.953 |
| ONATAZ | NPYD/N1 | OX/O1 | OX/O2 | OX/O3 | OX/O4 | NPYD/N1A | OX/O1A | OX/O2A | OX/O3A | OX/O4A |  |  |  |  |
|  | -0.017 | 0.020 | 0.035 | 0.031 | 0.045 | -0.017 | 0.019 | 0.035 | 0.030 | 0.044 | 0.031 | 0.518 | 2.889 | 2.884 |
| QATQAF | NPYD/N1 | OX/O2 | OX/O4 | OX/O3A | OX/O5A | OX/O3 | OX/O5 | NPYD/N1A | OX/O2A | OX/O4A |  |  |  |  |
|  | -0.031 | 0.016 | 0.014 | 0.045 | 0.029 | 0.043 | 0.034 | -0.031 | 0.018 | 0.009 | 0.029 | 0.841 | 2.903 | 2.853 |
| QETGAY | NPYD/N1 | OX/O1 | OX/O3 | OX/O4 | OX/O2B | OX/O2 | NPYD/N1B | OX/O1B | OX/O3B | OX/O4B |  |  |  |  |
|  | 0.004 | 0.030 | 0.019 | 0.029 | -0.002 | -0.002 | 0.004 | 0.029 | 0.018 | 0.028 | 0.020 | 0.470 | 2.959 | 2.862 |
| RUDWUJ | OX/O1 | OX/O2 | OX/O3 | OX/O4 | NPYD/N1 | OX/O1B | OX/O2B | OX/O3B | OX/O4B | NPYD/N1B |  |  |  |  |
|  | 0.055 | 0.026 | -0.005 | 0.012 | -0.012 | 0.055 | 0.026 | -0.005 | 0.012 | -0.012 | 0.028 | 0.775 | 2.931 | 2.858 |
| RUGVOF | NPYD/N3 | OX/O1 | OX/O2 | OX/O4 | OX/O5 | NPYD/N3A | OX/O1A | OX/O2A | OX/O4A | OX/O5A |  |  |  |  |
|  | -0.012 | 0.031 | 0.037 | 0.001 | 0.016 | -0.011 | 0.030 | 0.037 | 0.001 | 0.015 | 0.023 | 0.851 | 2.892 | 2.841 |
| SADDUY | NPYD/N1 | OX/O2 | OX/O5 | OX/O3B | OX/O4B | OX/O3 | OX/O4 | NPYD/N1B | OX/O2B | OX/O5B |  |  |  |  |
|  | 0.019 | 0.034 | -0.017 | 0.030 | -0.006 | 0.031 | -0.005 | 0.019 | 0.033 | -0.016 | 0.023 | 0.903 | 2.970 | 2.868 |
| TAHYEI | NPYD/N2 | OX/O1 | OX/O3 | OX/O5 | OX/O7 | NPYD/N1 | OX/O2 | OX/O4 | OX/O6 | OX/O8 |  |  |  |  |
|  | -0.009 | 0.000 | 0.052 | 0.000 | 0.030 | -0.012 | 0.020 | 0.022 | 0.032 | 0.002 | 0.024 | 0.383 | 2.905 | 2.893 |
| TAYFIJ | OX/O2 | OX/O3 | OX/O4 | OX/O5 | NPYD/N1 | OX/O2A | OX/O3A | OX/O4A | OX/O5A | NPYD/N1A |  |  |  |  |
|  | 0.102 | 0.043 | -0.020 | -0.040 | 0.019 | 0.060 | 0.025 | -0.001 | -0.021 | -0.001 | 0.044 | 0.363 | 2.922 | 2.916 |
| XAYKOY | OX/O1 | OX/O2 | OX/O3 | OX/O4 | NPYD/N1 | OX/O1A | OX/O2A | OX/O3A | OX/O4A | NPYD/N1A |  |  |  |  |
|  | 0.011 | 0.137 | -0.001 | 0.006 | -0.037 | 0.012 | 0.136 | -0.001 | 0.005 | -0.037 | 0.063 | 0.355 | 2.989 | 2.965 |

**Form of the electrostatic potential energy employed in MOE.**

*E*_ele_ is the electrostatics energy:


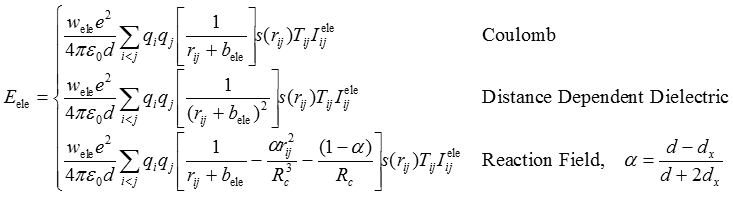


where *w*_ele_ is a weight, *d* is the dielectric constant in the interior of the solute, *d_x_* is the dielectric constant of the solvent, *s* and *T* are as in the van der Waals energy (see below), *q_i_* is the partial charge on atom *i*, *b*_ele_ is a buffering constant to prevent zero denominators, *R_c_* is the non-bonded cutoff distance. *I*^ele^, similarly to *I*^vdw^, is an interaction scale factor defined to be 0 for 1-2 and 1-3 interactions, a parameter set-dependent scale value for 1-4 interactions, and 1 for other interactions.

*s* is the smoothing (cutoff) function:


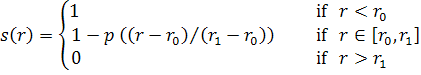


where *p*(*x*) = *x*^3^ (6*x*^2^ - 15*x* + 10). This polynomial has the properties that *p*(1) = 1, *p*(0) = 0, *p*'(0) = *p*'(1) = 0 and *p*''(0) = *p*''(1) = 0. By setting the cutoff parameters *r*_0_ and *r*_1_, a variety of smooth tapering functions that are continuous in both their first and second derivatives can be created.

*T_ij_* is an interaction scale factor used to scale particular non-bonded interactions. Associated with each atom *i* is a *state value* *T_i_*. This state value is an integer that can be set with the SVL function aSetState. Given two atoms *i* and *j*, the *T_ij_* scale factor is defined to be:


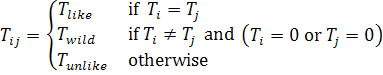


where T_like_, T_unlike_ and T_wild_ are state parameters which can be adjusted with the State Scale fields in the panel. For example, to disable interaction energies between two molecules, set the state *T_i_* of the atoms in the first molecule to 1 and the *T_i_* of the atoms in the second molecule to 2. Then set T_like_ to 1 and T_unlike_ to 0. In this way, only the inter-molecular non-bonded forces will be disabled, not the intra-molecular non-bonded forces. Note that a *T_i_* of 0 is like a wildcard in that it will match any other value.
